# Sphingosine-1-Phosphate Induces ATP Release via Volume-Regulated Anion Channels in Breast Cell Lines

**DOI:** 10.3390/life11080851

**Published:** 2021-08-19

**Authors:** Kishio Furuya, Hiroaki Hirata, Takeshi Kobayashi, Masahiro Sokabe

**Affiliations:** 1 Mechanobiology Laboratory, Nagoya University Graduate School of Medicine, Nagoya 466-8550, Japan; hhirata@med.nagoya-u.ac.jp (H.H.); msokabe@med.nagoya-u.ac.jp (M.S.); 2 Department Human Nutrition, Nagoya University Graduate School of Medicine, Nagoya 466-8550, Japan; 3 Department Physiology, Nagoya University Graduate School of Medicine, Nagoya 466-8550, Japan; takeshik@med.nagoya-u.ac.jp

**Keywords:** ATP release, LRRC8A, S1P, hypotonic stress, ATP imaging, breast cancer cell, W146, JTE013, VRACs, tumor microenvironment

## Abstract

High interstitial level of ATP and its lysate adenosine in the cancer microenvironment are considered a halo mark of cancer. Adenosine acts as a strong immune suppressor. However, the source of ATP release is unclear. We clarified the release of ATP via volume-regulated anion channels (VRACs) in breast cell lines using an ATP luminescence imaging system. We detected a slowly rising diffuse pattern of ATP release that was only observed in undifferentiated cells, not in differentiated primary cultured cells. This was confirmed by suppression with DCPIB, a blocker of VRACs, and shRNA for LRRC8A, an indispensable subunit of VRACs. We herein demonstrated that the inflammatory mediator sphingosine-1-phosphate (S1P), which exists abundantly in the cancer microenvironment, induced a diffuse pattern of ATP release isovolumetrically. The response was dose-dependent and suppressed by the knock-down of LRRC8A. It was also suppressed by blockers of S1P receptor 1 and 2 (W146 and JTE013, respectively). RTqPCR demonstrated the prominent presence of S1PR1 and S1PR2 mRNAs. We discussed the roles of S1P-induced ATP release in the cancer microenvironment.

## 1. Introduction

ATP is quickly hydrolyzed by ecto-ATPase in the extracellular space and is maintained at a concentration near zero in the interstitial fluids of unstressed tissues. This provides a condition under which ATP works as a ubiquitous extracellular signaling molecule through its instantaneous release. However, the extracellular ATP concentration is considerably high at sites of inflammation or in cancerous tissues [1,2], despite the abundance of ecto-ATPase. The functions of chronically high extracellular ATP are gradually being clarified. One important function is the production of adenosine by hydrolysis with ecto-ATPase, CD39 and CD73 and the maintenance of a high level of adenosine in the cancer microenvironment. Chronically increased levels of adenosine have been shown to contribute to the suppression of immune attack on tumor cells via multiple pathways, including the inhibition of T cells and dendritic cells [3]. Despite increasing evidence to support the importance of purinergic pathways in the cancer microenvironment, the source of ATP, which is itself a major source of adenosine, remains unclear.

**Figure 1:**
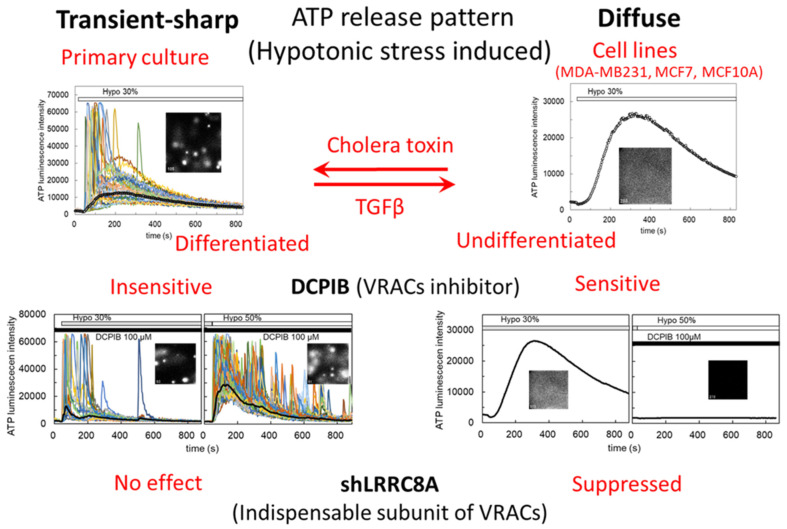
Two patterns of the ATP release induced by hypotonic stress in mammary epithelial cells in primary culture and in cell lines. This shows the outline of our preceding report [4]. Using our ATP luminescence imaging system, we found the following. (1) By hypotonic stress, ATP was intermittently released with transient-sharp peaks in primary culture of mammary cells, while in breast cell lines (MDA-MB231, MCF7, MCF10A), ATP was released with a slowly rising diffuse pattern. (2) The diffuse pattern changed to a transient-sharp one with cholera toxin treatment and the reverse change was induced by TGFβ treatment. Cholera toxin suppresses inflammation and carcinogenesis, and occasionally induces differentiation of various types of cells [5,6]. In contrast, TGFβ sometimes induces carcinogenesis and epithelial-to-mesenchymal transition (EMT) in both development and carcinogenesis [7,8]. (3) DCPIB, an inhibitor of VRACs, only suppressed the diffuse pattern. (4) Knockdown of LRRC8A, the essential molecular entity of VRACs, suppressed the diffuse pattern. These results suggest that abundantly expressed VRACs are a conduit of ATP release in undifferentiated cells, including cancer cells.

In our preceding paper [4], we studied the mechanisms underlying hypotonic stress-induced ATP release in mammary epithelial cells in primary culture and cell lines, using an ATP luminescence imaging system (Figure 1). We demonstrated that, in primary cultured cells, ATP was intermittently released with transient-sharp peaks, while in breast cell lines, ATP was released with a slowly rising diffuse pattern; the diffuse pattern changed to a transient-sharp pattern with cholera toxin treatment, and the reverse change was induced by TGFβ treatment; DCPIB (4-[(2-butyl-6,7-dichloro-2-cyclopentyl-2,3-dihydro-1-oxo-1h-inden-5-yl)oxy]butanoic acid), an inhibitor of volume-regulated anion channels (VRACs), only suppressed the diffuse pattern; knockdown of LRRC8A, the essential molecular entity of VRACs, also suppressed the diffuse pattern (Figure 1). These results suggest that abundantly expressed VRACs contribute to the release of ATP in undifferentiated cells including cancer cells.

VRACs are natively Cl^−^ channels composed of a heteromeric hexamer of LRRC8A to E five isoforms. They permeate various substances, including glycine, aspartate, glutamate, GABA, taurine, myo-inositol, lactate and ATP. These variations in the properties of VRACs are now considered to result from variations in the heteromeric subunit structures and correlated proteins in the LRRC8 hexamer [9,10,11,12,13,14], although the roles of each subunit remain unclear. VRACs work as a cell volume regulator and also participate in numerous physiological and pathophysiological processes, including, the cell cycle, proliferation, migration, apoptosis and cancer progression [15,16,17]. In our preceding study [4], we also found that sphingosine-1-phosphate (S1P), an inflammatory mediator richly present in the cancer microenvironment, induced the diffuse release of ATP isovolumetrically. S1P-induced ATP release was also suppressed by DCPIB treatment and in LRRC8A knockdown cells, indicating the involvement of VRACs. In the present study, we investigated the S1P-induced release of ATP in detail.

S1P is a signaling lysophospholipid involved in various cellular functions, including proliferation, migration, cytoskeletal rearrangement and adhesion [18]. S1P signaling also plays essential roles in several diseases, including inflammation, cancer and autoimmune disorders [19,20]. S1P is rich in the cancer microenvironment [21] and plays important roles in cancer progression via diverse pathways of its high-affinity G protein-coupled receptors (GPCRs), suggesting that the S1P pathway is a viable therapeutic target [22,23,24]. S1P binds to a family of five GPCRs: S1PR1 to 5, which are differentially coupled to heterotrimeric G proteins Gi/o, Gq and G12/13. Differential but overlapping expression patterns of S1PRs and coupled intracellular pathways of each receptor enable S1P to exert diverse functions. Thus, the response to S1P is totally cell context dependent. We herein report the involvement of S1PR1 and S1PR2 in the activation of ATP release via VRACs in various types of breast cell lines and discuss the roles of S1P-induced ATP release via VRACs in the cancer microenvironment.

## 2. Materials and Methods

### 2.1. Cell Culture

Breast cancer cell lines MCF7 (AKR-211, MCF-7/GFP; Cell Biolabs, Inc., San Diego, CA, USA) and MDA-MB231 (AKR-201, MDA-MB231/GFP; Cell Biolabs, Inc., San Diego, CA, USA) were cultivated in DMEM/F12 (Wako Pure Chemical, Osaka, Japan) supplemented with 10% FBS (Origen Mexico; Gibco, Thermo Fisher Scientific, Waltham, MA, USA). The non-carcinogenic breast epithelial cell line MCF10A (CRL-10317; ATCC, Manassas, VA, USA) was cultivated on a collagen-coated dish in HuMEC (Gibco, Thermo Fisher Scientific, Waltham, MA, USA) at 37 °C under 5% CO_2_. For measurements, cells were cultured on collagen gel (Cellmatrix type I-A; Nitta Gelatin, Osaka, Japan) on a 14- or 22-mmφ cover glass (#1; Matsunami Glass Ind. Ltd., Osaka, Japan) for 1–4 days at 37 °C under 5% CO_2_, in sub-confluent to confluent conditions. In some experiments, 3 types of cells (e.g., different cell lines or different shRNA treated cells) each on 3 or 6 separated collagen-gel patches were cultured simultaneously on a 22-mmφ cover glass, which made it easy to compare the responses under the same conditions of cultivation and measurement.

### 2.2. Experimental Setup

The cells on the cover glass were set in a small perfusion chamber (approximately 100 µL in volume) on the stage of an upright microscope (BX51WI; Olympus, Tokyo, Japan) with a 1× (Plan UW, NA0.04; Nikon, Tokyo, Japan) objective lens. The medium was replaced with DME/F12 buffered with 10 mM HEPES (pH 7.4) (Gibco) containing 40–50% luciferin-luciferase solution (see below). Medium changes (300 μL) were performed via capillary action for approximately 30 s without any mechanical effect of flow. Hypotonic solutions were made by adding a solution with 1.05 mM CaCl_2_ + 0.7 mM MgCl_2_ (30–50% [*v*/*v*]), which keeps the Ca^2+^ and Mg^2+^ concentration constant in hypotonic solutions. The osmolality of each medium as follows: DME/F12-HEPES, 303 mosm; 30% hypotonic solution, 218 mosm; 50%hypotonic solution, 155 mosm. Sphingosine-1-phosphate (Huzzah S1P, Human Serum Albumin/sphingosine-1-phosphate Complex; Avanti Polar Lipids, Inc., Alabaster, AL, USA), W146 ([(3R)-3-amino-4-[(3-hexylphenyl)amino]-4-oxobutyl]-phosphonic acid, TOCRIS, Bristol, UK) and JTE013 (1-[1,3-Dimethyl-4-(2-methylethyl)-1H-pyraazolo [3,4-b]pyridin-6-yl]-4-(3,5-dichloro-4-pyridinyl)-semicarbazide, Sigma-Aldrich, St. Louis, MO, USA) were added to the medium with luciferin-luciferase. The medium containing agonist or antagonists were applied to the small perfusion chamber via capillary action.

### 2.3. Real-Time Imaging of the ATP Release

The ATP release was measured in real-time using a luminescence imaging system that has been previously described [25]. Luciferin-luciferase ATP bioluminescence was detected using a high-sensitivity camera system simultaneously with infrared imaging to monitor the cells. An osmolality-adjusted luciferin-luciferase solution (Luciferase FM plus; Bioenex Inc., Hiroshima, Japan) was added to the perfusate at 40–50% volume. After being left standing for 15 min after a medium change, ATP-dependent luminescence was detected with a high-sensitivity EMCCD camera (Cascade 512F; Photometrics, Tucson, AZ, USA) equipped with a cooled image intensifier (C8600-04; Hamamatsu Photonics, Hamamatsu, Japan). Images were acquired at a frequency of 1 or 2 Hz with an exposure time of 1s or 500 ms using the MetaMorph software program (ver. 7.8; Molecular Devices, San Jose, CA, USA) in stream acquisition mode. For the data analyses, image smoothing was usually conducted by calculating the average of four to six sequential images. The region of interest (ROI) was set at the cell colony, and the average intensity of the ATP luminescence in the ROI was measured over time. This value is related to the concentration of ATP [25]. ATP imaging experiments were performed at 30 ± 2 °C.

### 2.4. Quantitative Reverse Transcription Polymerase Chain Reaction (RTqPCR)

The expression of S1P receptor family members S1PR1 to 5 was measured by RTqPCR. mRNA specimens were isolated from cells grown on collagen-gel in 24-well dishes using a NucleoSpin RNAplus RNA isolation kit (Macherey-Nagel, Dueren, Germany) and converted to cDNA with SuperScript IV VILO Master Mix (Invitrogen, Thermo Fisher Scientific, Waltham, MA, USA). The expression was determined by RT-qPCR using a LightCycler 480 (Roche, Mannheim, Germany) with SYBR Green I Master Mix (Roche, Mannheim, Germany) and quantitative primers (Perfect Real Time Primer; Takara, Shiga, Japan, Table 1). The expression was normalized to GAPDH within each sample.

### 2.5. LRRC8A Knockdown with shRNA

LRRC8A silencing with shRNA was performed using retrovirus mediated gene transfer as described previously [4,26]. To generate retrovirus expressing shRNA against LRRC8A, the target sequences (Table 1) were inserted into the pSUPER.retro.puro retroviral vector (OligoEngine, Seattle, WA, USA). As a control, a non-targeting sequence 5′-ATAGTCACAGACATTAGGT-3′ was introduced. Cloned cell lines stably expressing shLRRC8A showed prominent suppression of LRRC8A mRNA but almost no effects on the expression of other LRRC8 isoforms (LRRC8A-E) in three breast cell lines (Supplemental Figure S1).

## 3. Results

### 3.1. Hypotonic Stress-Induced ATP Release via VRACs

**Figure 2:**
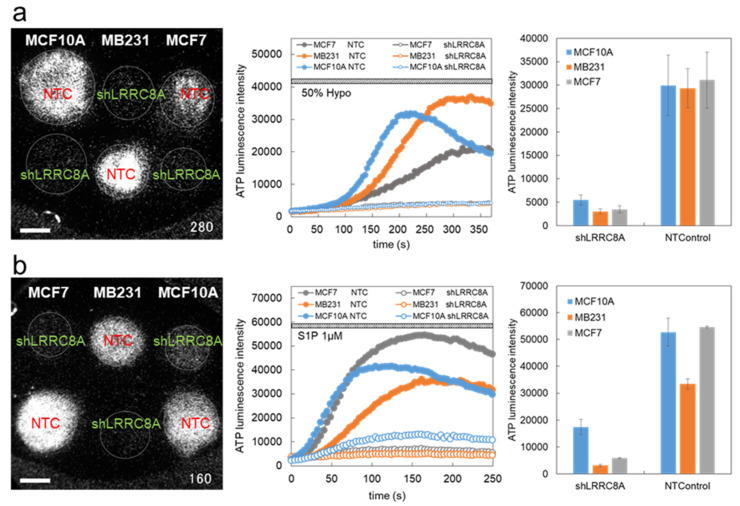
The diffuse release of ATP induced by hypotonic stress (**a**) and S1P (**b**) in LRRC8A knockdown cells and NTControl cells in three breast cell lines (MCF10A, MCF7, MDA-MB231). (**a**): A typical response to hypotonic stress (50%) is shown in the left panel. See also Supplemental Video S1. The time courses of the ATP luminescence change in each colony of cells are shown in the center panel. The peak intensity of ATP luminescence in each colony was measured and is plotted in the right panel. Scale bar is 2 mm. Values represent the mean ± S.E. (N = 4–9). In this experiment, 50% hypotonic solution was applied after certain measurements using 30% hypotonic solution; thus, the practical change in hypotonicity in this data was 28.6% (1 − (1 − 0.5)/(1 − 0.3)). (**b**) A typical response to S1P (1 µM) is shown in the left panel. See also Supplemental Video S2. The time courses of the ATP luminescence change in each colony of cells are shown in the center panel. The peak intensity of ATP luminescence in each colony was measured and is plotted in the right panel. Scale bar is 2 mm. Values represent the mean ± S.E. (N = 3). In this experiment, S1P was applied after 5.5 min of pre-stimulation with 30% hypotonic solution, which remarkably enhanced the response to S1P [4].

We used three breast cell lines—MDA-MB231, MCF7 and MCF10A—with two conditions of gene knockdown in each cell line, shLRRC8A and non-targeting control (NTControl). For efficient measurement, we simultaneously cultured three types of cell lines under two conditions on six separate collagen-gel patches on a cover glass and measured the ATP luminescence of each cell colony at the same time (Figure 2a). When hypotonic stress (50%) was applied, slowly rising diffuse ATP release was observed in NTControl cells but not in LRRC8A knockdown cells in each breast cell line (Figure 2a, Supplemental Video S1). As shown in the preceding paper [4], this result indicated that VRACs contributed to diffuse ATP release by hypotonic stress in the breast cell lines. Among the three cell lines the responses to hypotonic stress were not markedly different, although some variations in kinetics and amplitude were seen. The pattern of ATP response to hypotonic stress mainly changed with the state of the cells in culture (i.e., in cholera toxin-treated differentiated cells or in primary cultured cells, the diffuse pattern of ATP release via VRACs was reduced and an intermittent sharp pattern of ATP release appeared) (Figure 1, [4]).

### 3.2. S1P-Induced ATP Release via VRACs

**Figure 3:**
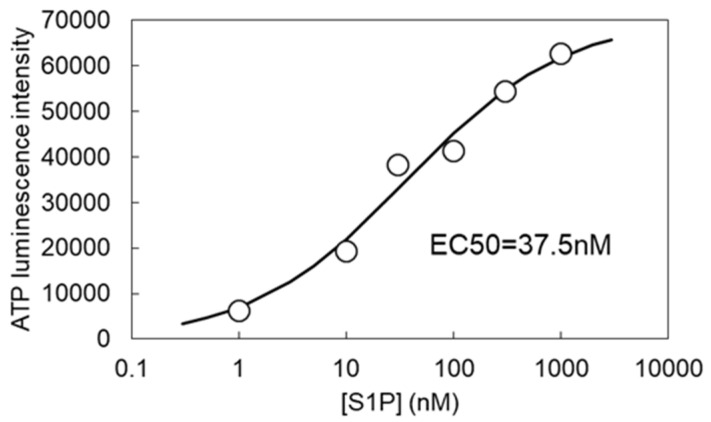
The dose dependence of S1P on the ATP release. The release of ATP induced by each concentration of S1P was measured in MCF7 cells. S1P was applied after 5.5 min of pre-stimulation with 30% hypotonic solution to enhance the response to S1P [4]. The sigmoidal fitting curve was calculated using the Origin software program (OriginLab Corp, Northampton, MA, USA). The obtained EC_50_ was 37.5 nM.

In addition to hypotonic stress, VRACs are isovolumetrically activated by various substances, including the inflammatory mediator sphingosine-1-phosphate (S1P). The application of S1P (1 µM) induced slowly rising diffuse ATP release in NTControl cells, which was notably suppressed in LRRC8A knockdown cells of each breast cell line (Figure 2b, Supplemental Video S2). The time courses of ATP release showed that the initiation of the ATP release by S1P occurred faster than that by hypotonic stress. There was also some variation in the kinetics and peak intensity among the three cell lines; however, a common feature was the significant suppression of the ATP release in LAAC8A knockdown cells, suggesting the contribution of VRACs to the S1P-induced release of ATP. We measured the dose-dependence of S1P on the ATP-release in MCF7 cells (Figure 3). Even with 1 nM S1P, a clear response of ATP luminescence was observed. A fitting curve calculated with sigmoidal fitting showed that the EC50 was 37.5 nM. In these experiments, S1P was applied after approximately 6 min of pre-stimulation with 30% hypotonic solution, which remarkably enhanced the response to S1P in comparison to the sole administration of S1P, as shown in the preceding paper [4].

### 3.3. Subtypes of S1P Receptors

**Figure 4:**
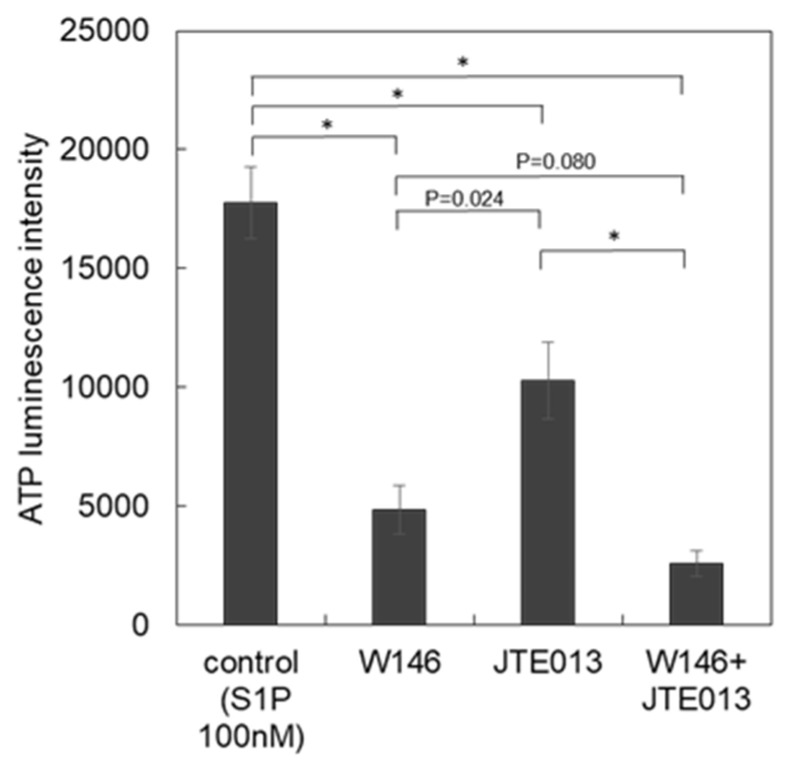
Blocking effects of W146 and JTE013 on the release of ATP with S1P. W146 and JTE013 are antagonists for S1PR1 and S1PR2, respectively. The ATP response to 100 nM S1P was measured in the presence or absence (control) of antagonists (W146 100 μM, JTE013 100 μM and both). S1P was applied following 6 min of 30% hypotonic pre-stimulation, which contained each antagonist. The peak intensity around several minutes after the S1P application was measured. Each antagonist partially blocked the ATP release while the use of both antagonists suppressed the ATP release further. The data in three cell lines were averaged. Values represent the mean ± S.E. (N = 15, 7, 8, 8, from the left to the right), *t*-test; *: *p* < 0.001.

**Figure 5:**
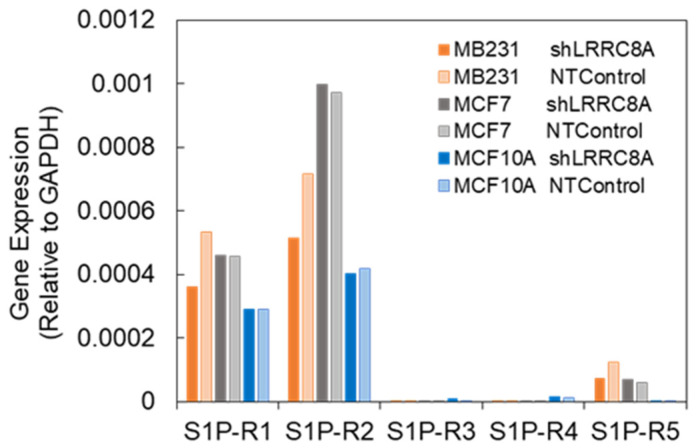
Subtypes of S1P receptor mRNAs appeared in three breast cell lines. RTqPCR of S1PR1 to R5 was performed in LRRC8A knockdown and NTControl cells in each cell line. S1PR1 and R2 were prominent and the knockdown of LRRC8A had no effect on the S1PR expression.

S1P is known to activate various intracellular signaling cascades through its G protein-coupled receptors 1–5. To elucidate the subtypes of S1P receptors involved in VRAC activation, pharmacological and RT-qPCR experiments were performed. W146 and JTE013 are relatively specific blockers for S1PR1 and S1PR2, respectively [18]. The ATP release induced by S1P (100 nM) was measured in the absence (control) or the presence of blockers (W146 and JTE013 at 100 µM each or together), and the peak intensity of each response was plotted (Figure 4). The blockers were applied 6 min prior to S1P application. The application of blockers at 100 µM each or together did not induce any ATP release, suggesting no cytotoxicity, such as cell lysis, in the short term, although the long-term effect on the cell growth or metabolisms is unclear. Each antagonist partially blocked the ATP release induced by S1P and the application of both in combination blocked the ATP release further. This result suggested that both the S1PR1 and S1PR2 receptors contribute to the activation of VRACs. To confirm this, RTqPCR for S1PR1 to R5 was performed for the three breast cell lines that expressed shLRRC8A or shNTControl (Figure 5). In all three cell lines, mRNAs of S1PR1 and S1PR2 were prominently appeared but S1PR3 to R5 were obscure. The knockdown of LRRC8A did not affect the expression of S1PRs. These results suggested that, in these breast cell lines, S1P activated VRACs through S1PR1 and S1PR2.

## 4. Discussion

In addition to the preceding paper [4], we showed here that S1P induced the diffuse release of ATP in a dose-dependent manner, suggesting the involvement of its receptors S1PR1 and R2 in three breast cell lines. This induction was suppressed by the knockdown of LRRC8A, suggesting that VRACs contributed to the diffuse release of ATP. How S1P activates VRACs is unclear. Extracellular S1P binds to S1PR1 to R5, which are differentially coupled to heterotrimeric G proteins. S1PR1 couples to Gi/o, while S1PR2 may couple to Gi/o, Gq, or G12/13 [18]. They are considered to be crucial regulators of the cancer cell growth and survival via the activation of key pathway clusters, such as Ras/Erk and PI3K/Akt [18,22]. It was reported that S1P-induced release of ATP via VRACs formed an autocrine link between inflammatory sphingolipid and purinergic signaling in RAW macrophages [27] and BV-2 microglia [28]. In these cells, it was suggested that Gi/o-coupled S1PR1 and rearrangement of the actin cytoskeleton were involved in VRAC activation with S1P. VRACs were reported to be isovolumetrically activated or modulated by a numerous signaling molecules, including intracellular GTPγS, Rho and Rho kinase (ROCK), phosphatidyl-inositol-3-kinase (PI3K), tyrosine kinases, purinergic signaling, bradykinin, mGluR, ROS and Ca^2+^ signaling [16,29,30]. Matching the S1PR signaling cascade to the mechanism of VRAC activation should induce the VRACs to release ATP with S1P. Confirmation of the receptor subtypes and the investigation of the signaling cascade coupled to VRAC activation are issues to be addressed in the next step. From a structural perspective, the leucine-rich repeat (LRR) domains in the LRRC8 hetero hexamer, which forms a dome structure at the cytoplasmic side of the membrane, are considered essential for the volume sensitivity and interaction with various functional proteins as a scaffold protein [31].

VRACs were also reported to be activated by the unfolding of membrane reserves, like caveolae, upon cell swelling, which enables VRACs to interact other functional proteins, including actin cytoskeleton [32,33,34]. Interestingly, the ATP response to S1P was enhanced under hypotonic conditions, suggesting that the activation of S1PR also depended on the unfolding of the membrane [4]. It is plausible that VRACs and S1PR interact through membrane dynamics, such as via VRACS and the anoctamin 1 Ca^2+^-activated Cl^−^ channel [33]. Recently, VRAC-dependent ATP release was reported to be controlled by ABC subfamily G member 1 (ABCG1; a cholesterol transporter) using gain-of-function screening [35]. It was shown that reducing cellular cholesterol levels stimulated VRAC-dependent ATP release. While not mentioned this result also implied the contribution of caveolae to VRAC activation.

In the present and preceding studies [4], we used three breast cell lines that originated from different tissue states. MDA-MB231 cells are derived from adenocarcinoma and are highly aggressive with triple-negative properties [36]. MCF7 cells are a ductal carcinoma cell line with an estrogen-sensitive property [37]. MCF10A cells originate from benign tumors of fibrocystic disease and are non-carcinogenic, although not normal karyotypically [37]. These cell lines possess different characteristics, as shown in their gene and protein expression profiles [38]. The subtypes of S1P receptors also differentially expressed among these cell lines and change with the cell state [39]; for example, tamoxifen treatment altered the expression of S1PR3 to S1PR2 [40]. However, in our experiments, S1P induced a similar response of ATP release in all of these cell lines, and the gene expression patterns of S1PRs were similar (i.e., S1PR1 and S1PR2 were prominently expressed). The response of ATP release to hypotonic stress and the expression pattern of the LRRC8A subfamilies were also similar among these cell lines [4]. These cell lines have different origins and properties; however, all are immortal and show undifferentiated properties in usual culture conditions. We hypothesized that the appearance of diffuse ATP release via VRACs depends on the undifferentiated state of the cells, including cancer cells.

Cytosolic ATP lies in a state of dynamic balance between production and consumption. Under the Warburg effect, ATP production by aerobic glycolysis in cytosol becomes the major rout of production in cancer cells, while production via oxidative phosphorylation in mitochondria is prominent in normal cells [41,42]. The Warburg effect is considered a hallmark of not only cancer cells but also rapid proliferation of non-cancerous cells [43]. The release of ATP is influenced by cellular metabolism; however, whether or not the metabolic pathway is related to the release of ATP is unclear. In our preceding study [4], we demonstrated that the pattern of ATP release was changed from transient-sharp to diffuse by TGFβ treatment and the reverse change was induced by cholera toxin treatment even in the same cells (Figure 1). TGFβ induced carcinogenesis and EMT (epithelial-to-mesenchymal transition) [7,8] as well as Warburg-like cancer metabolism [44,45]. The administration of cholera toxin suppressed inflammation and carcinogenesis, and occasionally induced differentiation in various types of cells [5,6]. Activators of cAMP elicited an anti-Warburg effect [46]. These results suggest that the ATP release pattern (diffuse/transient-sharp) is related to the state of cells (undifferentiated/differentiated) as well as the metabolic pathways (glycolytic/oxidative). This is an interesting hypothesis should be explored in further studies.

**Figure 6:**
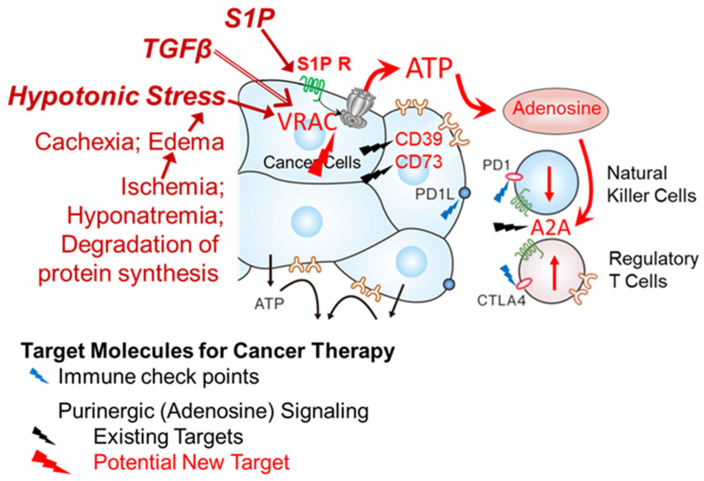
A schematic illustration of the hypothesized roles of ATP release in the cancer microenvironment. The slow and diffuse, but substantial and prolonged release of ATP via VRACs in cancer cells forms high-ATP interstitium around the cancer. ATP is degraded by ecto-ATPase CD39 and CD73, which are abundantly expressed in cancer cells, to produce adenosine. Adenosine suppresses the immune attack on the cancer through multiple pathways, including the suppression of natural killer cells and the activation of regulatory T cells via the A2A adenosine receptor. The activation of VRACs to release ATP is induced by hypotonic stress and S1P, an inflammatory mediator that exists abundantly in the cancer microenvironment. S1P activates its G-protein coupled receptors. Regarding hypo-osmotic stress, cells experience frequent fluctuations in volume due to unbalanced transmembrane fluxes of ions and nutrients or the synthesis and degradation of macromolecules within the cells. In cancer patients, the degradation of protein synthesis, including albumin, causes cachexia and edema. Thus, hypotonic stress may ordinarily occur in the cancer microenvironment. TGFβ is a major tumor progressor with EMT and also abundant in the cancer microenvironment. We found that TGFβ increased the VRAC expression and enhanced the release of ATP by both S1P and hypotonic stress [4]. The adenosine pathway was reported to synergistically interact with the immune check point pathway molecules (PD1, PD1L, CTLA4, etc.), which are strong target molecules [47]. CD39, CD73 and A2A are now considered molecular targets for cancer therapy [3]. Based on our results, VRACs or the source of the ATP signaling may be potential new therapeutic targets.

Figure 6 shows a schematic illustration of the hypothesized roles of ATP release in the cancer microenvironment. The high interstitial ATP concentration in the cancer microenvironment plays various roles in cancer. One important role is in the production of adenosine by ecto-ATPase (CD39, CD73). Adenosine is known to act as a strong suppressor of the immune attack on cancer via multiple pathways (e.g., suppression of natural killer cells and activation of regulatory T cells via adenosine receptor A2A). The continuous existence of adenosine means the continuous release of ATP from the cells, the mechanism of which remains unclear at present. We found that abundantly expressed VRACs contributed to the release of ATP in undifferentiated cells, including cancer cells. VRACs were activated by not only hypotonic stress but also S1P, which is rich in the cancer microenvironment. S1P activates VRACs through its G protein-coupled receptors, although the mechanism has not been elucidated. The activity or expression of VRAC was enhanced by treatment with TGFβ [4], which exists abundantly at tumor sites and plays central roles in carcinogenesis [8]. We therefore hypothesize that VRACs contribute to the ATP release in the cancer microenvironment, implying the suitability of VRACs or the source of purinergic signaling as a new therapeutic target.

## 5. Conclusions

S1P, an inflammatory mediator, is abundant in the cancer microenvironment and plays various roles in cancer progression via its G protein coupled receptors. We demonstrated that S1P induces the release of ATP via VRACs in undifferentiated breast cell lines. This may have led to the abundance of ATP in the cancer microenvironment. Its hydrolysis product adenosine works as a suppressor of the immune attack on the cancer. ATP-releasing mechanics may be a potential therapeutic target.

## Figures and Tables

**Table 1:** RT-qPCR Primers and shRNA Target Sequences.

**RT-qPCR Primers**			
**Target**		**Sequence(5′-3′)**	**Oligo Name (TaKaRa)**
S1PR1	F	GGCTATGTTGAGTACGTAGGCTGTG	HA258013-F
	R	TCCCGCTTACATGGAAACTTTG	HA258013-R
S1PR2	F	ATGCAAGGCGCAACTTGAGA	HA264697-F
	R	CTGCAGGTGTGGAGCTGAGAA	HA264697-R
S1PR3	F	AAAGCCCTAACCTTGAAGTTTGGAA	HA178037-F
	R	CAATCCCATCACATGGACTACGA	HA178037-R
S1PR4	F	TCGCTCAGCTTTCGGATG	HA139390-F
	R	ATCCACACGCAAGACTGCAA	HA139390-R
S1PR5	F	AACCGGCTGCAGACTGACAC	HA203213-F
	R	TGCACCTTTGGCTGCATTTC	HA203213-R
GAPDH	F	GCACCGTCAAGGCTGAGAAC	HA067812-F
	R	TGGTGAAGACGCCAGTGGA	HA067812-R
**shRNA Target Sequences**			
LRRC8A		GAGCGCAGTATTTGGATAA	shA

## Data Availability

Data are available from the corresponding author upon specific re-quest.

## Supplementary Materials

The following are available online at https://www.mdpi.com/article/10.3390/life11080851/s1, Video S1: The release of ATP induced by 50% hypotonic stress was suppressed in LRRC8A knock-down cells (shLRRC8A) but not in control cells (NTC) in each breast cell line; Video S2: The release of ATP induced by S1P (1 μM) was suppressed in LRRC8A knock-down cells (shLRRC8A) but not in control cells (NTC) in each breast cell line; Figure S1: Gene silencing with shRNA for LRRC8A in three breast cell lines.
