# Supplementary material for: Sphingosine-1-Phosphate Induces ATP Release via Volume-Regulated Anion Channels in Breast Cell Lines
Source: Life (Basel). 2021 Aug 19;11(8):851. doi: 10.3390/life11080851 (PMC8401269; doi:10.3390/life11080851)
Supplement: Supplementary file 1 [file life-11-00851-s001.zip › Supplemental Figure.pdf]

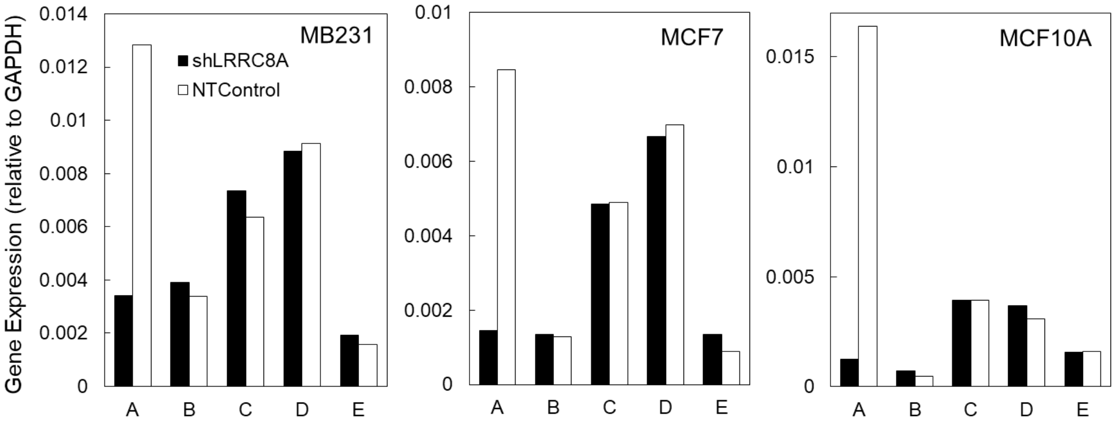

Figure S1. Gene silencing with shRNA for LRRC8A in three breast cell lines and suppression of the regulatory volume decrease (RVD) in knock-down cells. a, Nockdown of LRRC8A by shRNA (shA) suppressed the LRRC8A gene expression but did not affect the expression of other LRRC8 isoforms (LRRC8B to E) in three breast cell lines (MDA-Mb231, MCF7, MCF10A). The expression of LRRC8 isoforms (A, B, C, D, E) in the cells treated with non-targeting control (NTControl) and shA were measured by RT-qPCR and normalized to the GAPDH within each sample.
